# Patella fractures treated with suture tension band fixation

**DOI:** 10.1186/s13018-021-02309-5

**Published:** 2021-03-09

**Authors:** J. Adjal, I. Ban

**Affiliations:** Department of Orthopaedic Surgery and Traumatology, Hvidovre Hospital, CORH (Clinical Orthopaedic Research Unit), Hvidovre and Amager University Hospitals, Kettegaard Alle 30, Hvidovre, Capital Region 2650 Denmark

**Keywords:** Patella, Fracture, Patella fracture, Osteosynthesis, Suture, Suture tension band fixation

## Abstract

**Background:**

Patella fractures requiring surgery are traditionally treated using metallic implants, which are associated with high re-operation rates, mainly due to implant prominence. To overcome the problem of prominent metallic implants, we present a technique based purely on braided sutures.

**Methods:**

This technique is described in a step-wise, standardised way based on our findings on six patients treated at our institution.

**Results:**

This technique can be adapted to all types of patella fractures. The described suture configuration allows maintenance of inter-fragmentary reduction until bony union without symptoms from the suture material.

**Conclusions:**

We believe that this technique is a safe and promising alternative to traditional metallic fixation methods.

## Background

Fractures of the patella account for about 1% of all fractures in adults [1].

Patella fracture is commonly caused by either direct trauma (e.g. simple fall onto the knee or motor vehicle injuries) or indirectly following a forceful contraction of the quadriceps with the knee in a flexed position [2]. The fracture pattern can be transverse, vertical or stellate [3].

The extensor mechanism of the knee is crucial for ambulation. The patella is a sesamoid bone embedded in the quadriceps/patella tendons that displaces the tendons away from the centre of rotation, thereby increasing the moment arm and increasing the force of knee extension. The pull of the quadriceps tendon makes stable reconstruction of patella fractures a major surgical challenge [4–6].

The indications for surgical fixation of a patella fracture are an absent extensor mechanism (patient not able to lift leg straight), articular displacement >2 mm, or interfragmentary displacement >3 mm [7, 8]. Regardless of surgical technique, the goals of surgical treatment are the anatomical reduction of the articular surface, stable fixation able to withstand the deforming forces acting on the patella during the healing process and the preservation of a functional extensor apparatus to start early range of motion [1, 2, 9, 10].

Various fixation methods or modifications of previous methods have been described for the internal fixation of patella fractures. The modified tension band wiring technique using either Kirschner wires (k-wires) or screws perpendicular to the fracture with an anterior figure-of-eight metallic cerclage wire is probably the most commonly used [2, 9, 10]. High complication rates are common in most metal implants and secondary surgery for removal of prominent implants is often required. The reported rate for secondary implant removal following tension band wiring is 30–52% [11–14].

To overcome the problem of prominent implants without compromising stable fixation, we describe a standardised method for the fixation of simple transverse and comminuted patella fractures using non-absorbable, high-strength braided sutures applied only in soft tissue.

## Methods

All patients gave their informed consent upon receiving treatment with this technique. Patients were likewise informed of the possibility to be operated with tension band wiring with k-wires and cerclage. All patients accepted suture tension band fixation. The treatments of these six patients with the suture tension band fixation technique falls within the intern quality control assessment protocol of our institution.

### Surgical technique: indication, set-up, approach and reduction

This technique can be used for all types of acute patella fractures, including simple transverse, comminuted and pole fractures, as long as it is technically possible to reduce the fragments. The surgical steps differ according to the fracture pattern, as described below.

**Fig. 1:**
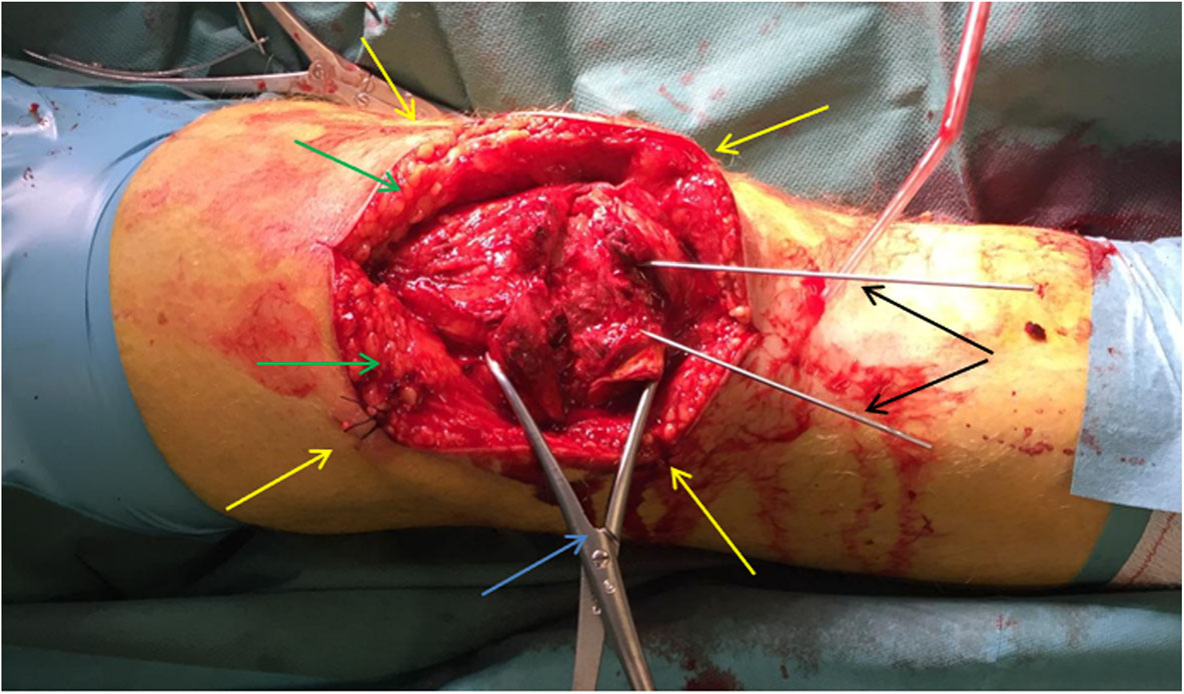
Intra-operative reduction of a simple transverse fracture. Green arrows: deep skin and subcutaneous flaps. Yellow arrows: holding sutures to tie the flaps down. Blue arrow: reduction clamp used initially for reduction. Black arrows: diverging k-wires inserted to secure the reduction and replace the reduction clamp. In colours

The patient is first placed supine on a radiolucent table. Prophylactic antibiotics are administered according to local guidelines. A tourniquet is not used. A longitudinal midline incision is made from approximately 5 cm above to 5 cm below the patella. Two deep skin and subcutaneous flaps are created and held in place with holding sutures to avoid retractor use (Fig. 1). All contused soft tissue is removed with care taken not to undermine the skin. The retinaculum overlying the fracture (often torn at the time of injury) is debrided and incised to identify all fracture fragments. All fracture lines are opened and meticulously cleared of debris. The fragment edges are identified by removing a few millimetres of soft tissue to aid in anatomical reduction. Fragment by fragment, the patella is reconstructed using a reduction clamp that eventually is replaced by k-wires. Medial and lateral longitudinal arthrotomies are performed to manually assess the articular surface reduction.

### Surgical technique: suture tension band (STB) fixation

**Fig. 2:**
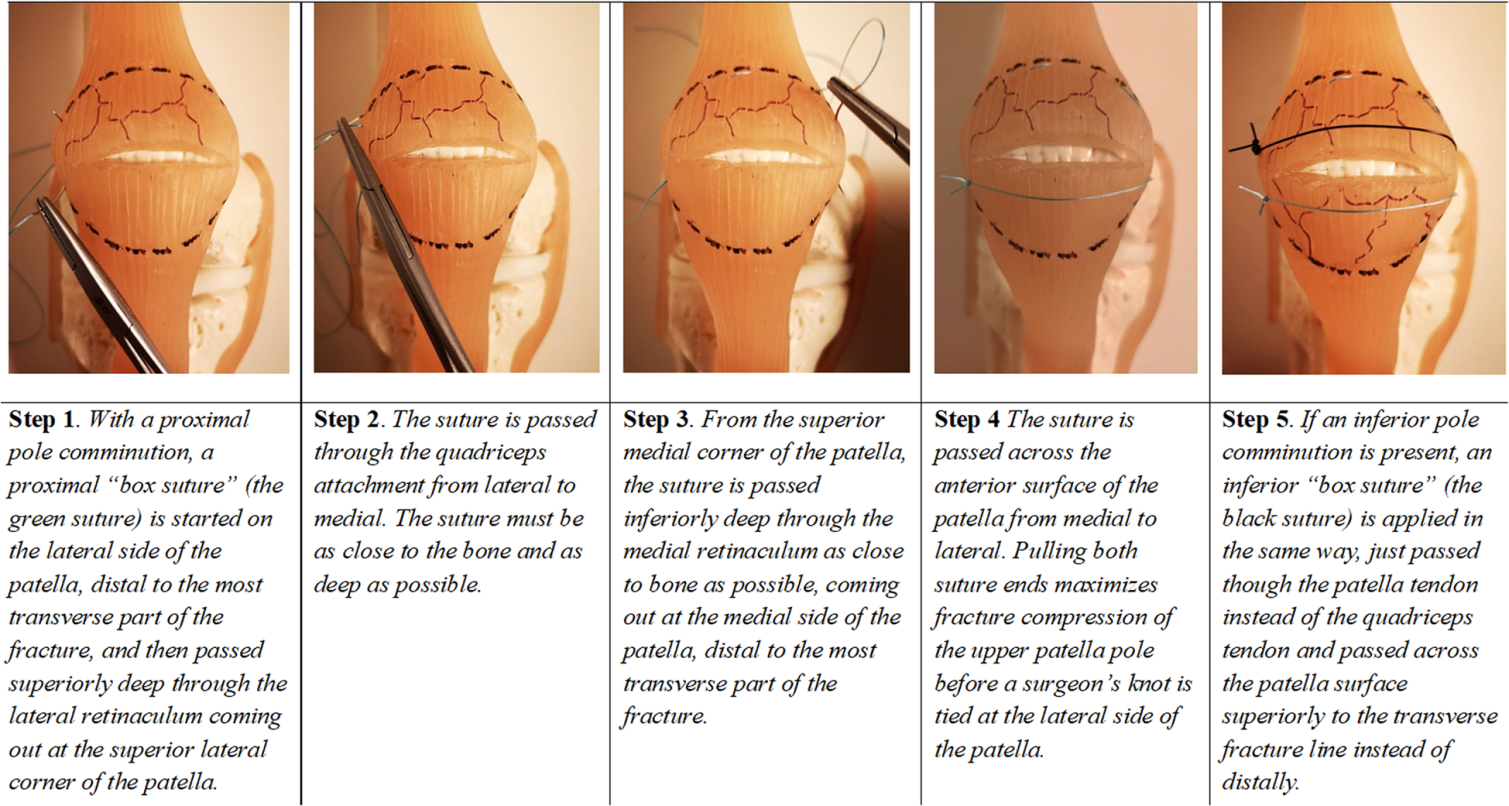
A step-wise description of the ‘box sutures’. In colours

For STB fixation, 2–4 FiberWire®#5 (Arthrex, Naples, FL, USA) are used depending on the fracture pattern as the STB technique differs for comminuted versus simple transverse fractures. If the fracture is comminuted at one or both ends of the patella, a ‘box suture’ that converts the fragments to a ‘single’ pole fragment is added to either one or both ends of the patella, depending on the fracture pattern (Fig. 2).

**Fig. 3:**
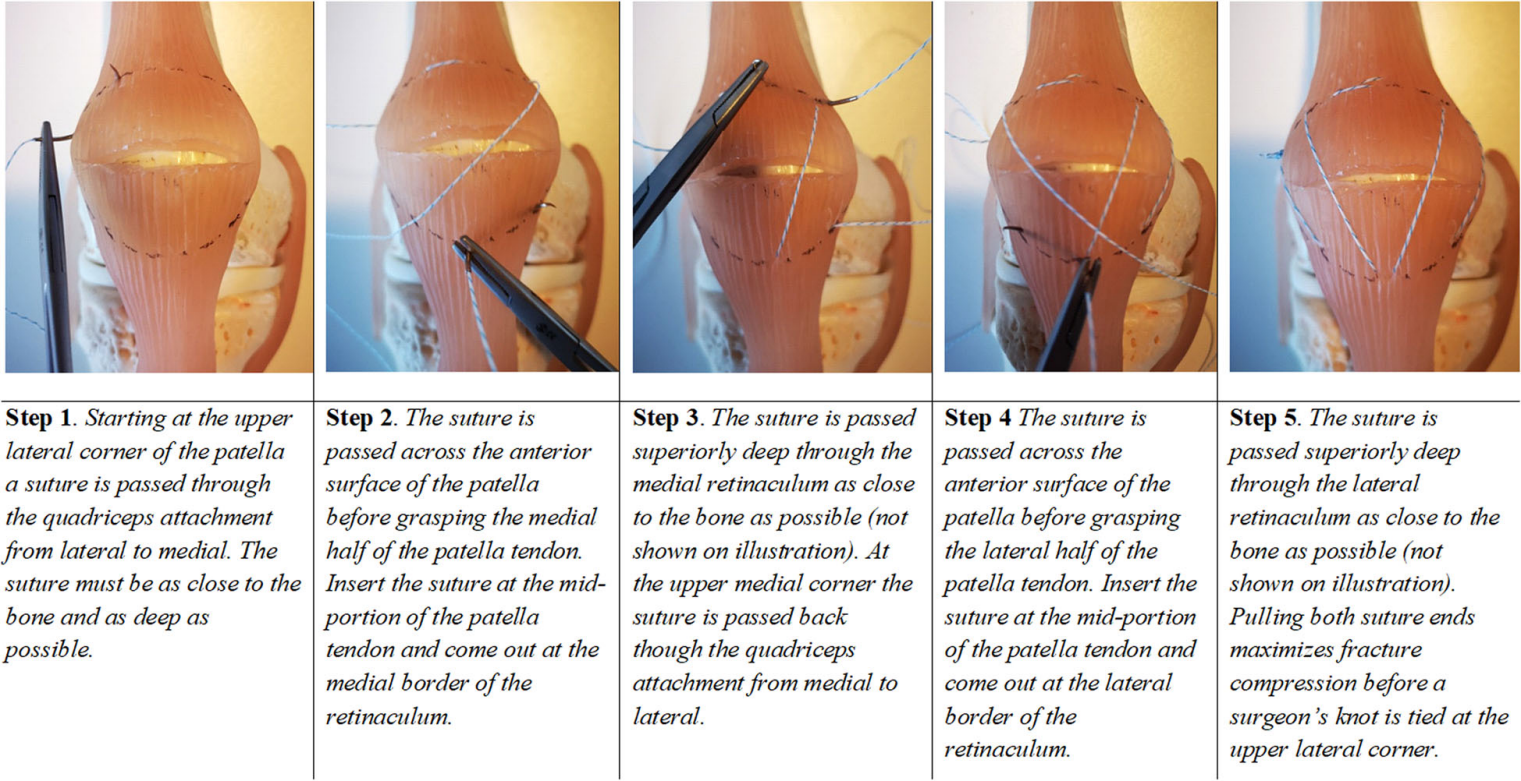
A step-wise description of the ‘modified circular suture’. In colours

**Fig. 4:**
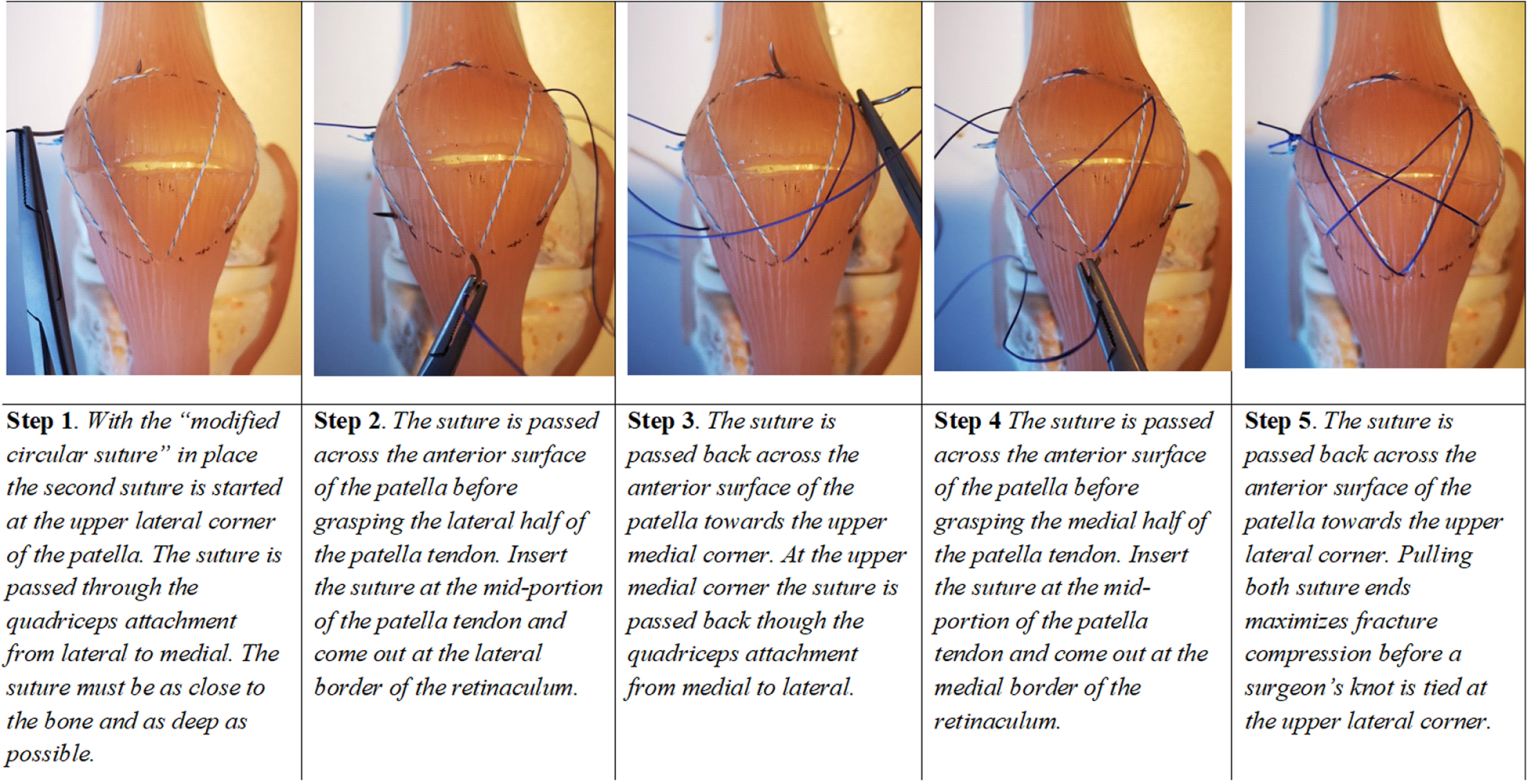
A step-wise description of the ‘modified figure-of-eight suture’. In colours

Thus, the comminuted fracture has been converted to a simple transverse fracture pattern. Following this step, the treatment of comminuted fractures is the same as that for simple transverse fractures, with fixation consisting of a ‘modified circular suture’ (Fig. 3) followed by a ‘modified figure-of-eight suture’ (Fig. 4).

**Fig. 5:**
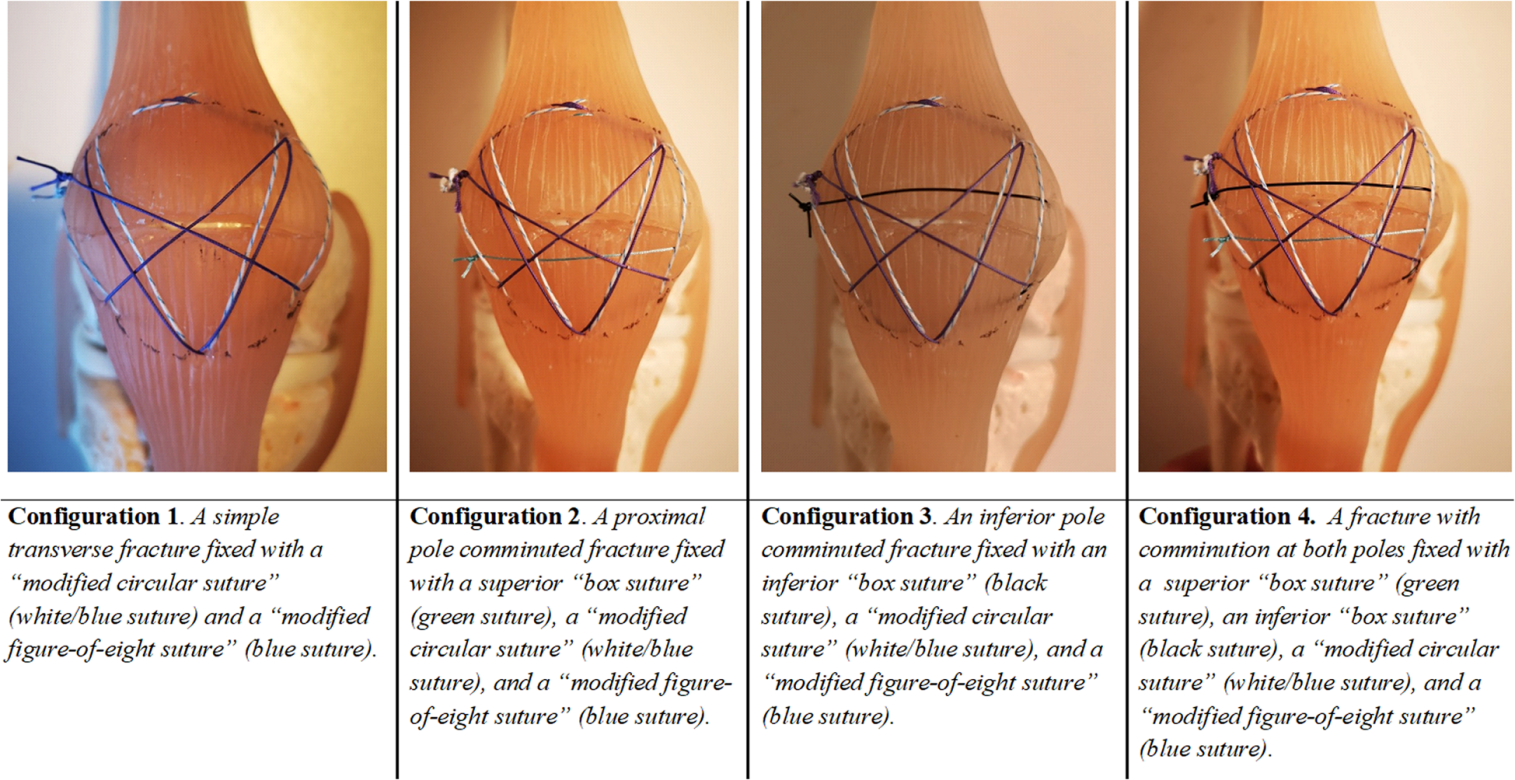
Illustration of the four different configuration options depending on fracture type. In colours

The different fixation configurations of transverse and comminuted fractures are illustrated in Fig. 5. During suture application, the k-wires are individually removed for optimal fragment compression.

The stability of the fixation is tested with ten cycles of manual knee bending (0–120 degrees). We believe that ten cycles are adequate in order for the suture to position itself intraoperatively. Reduction is confirmed by fluoroscopy before closing the arthrotomies and defects in the medial and lateral retinacula. Subcutaneous closure is performed with single inverted absorbable sutures before skin closure with staples.

### Surgical technique: postoperative management

The knee is initially immobilised with a removable knee-brace that allows an active range of motion (ROM) of 0–40 degrees and full weight-bearing. Before discharge, patients receive instruction on static quadriceps exercises and, if needed, are provided crutches. Two weeks postoperatively, the staples are removed, the wound is evaluated, and the knee brace is adjusted to allow a ROM of 0–60 degrees. At 4 weeks postoperatively, the knee brace is removed and the patients are encouraged to aid using the arms when the knee is bent at more than 90 degrees; otherwise, free ROM and weight-bearing can be performed as tolerated. At this point, the patients are referred to a physiotherapist for further muscle and ROM rehabilitation. At 12 weeks postoperatively, the patients are allowed movement and weight-bearing without restrictions.

## Clinical results

Between November 23, 2018, and January 30, 2019, six patients (two women and four men) with a median age of 61 years underwent the suture tension band fixation technique performed by the senior author. The patients were consecutively included regardless of fracture pattern or age as long as they were not able to extend against gravity or had a fracture with a displacement (including articular step-off) >2 mm. No patients were excluded.

**Fig. 6:**
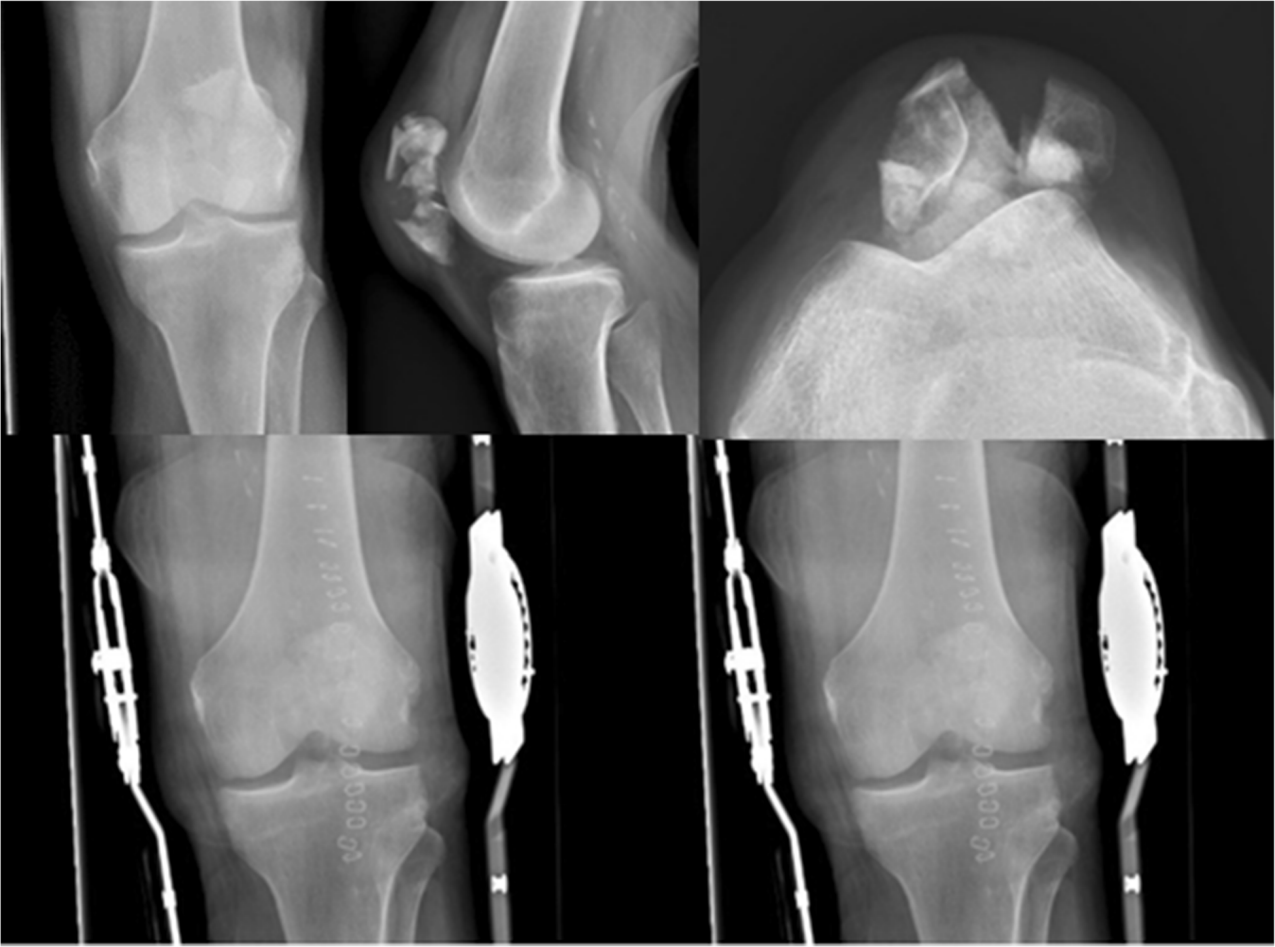
Pre- and postoperative X-rays (left and right, respectively) of patient 2, a 68-year-old man who presented with a severely comminuted left-side patella fracture

**Fig. 7:**
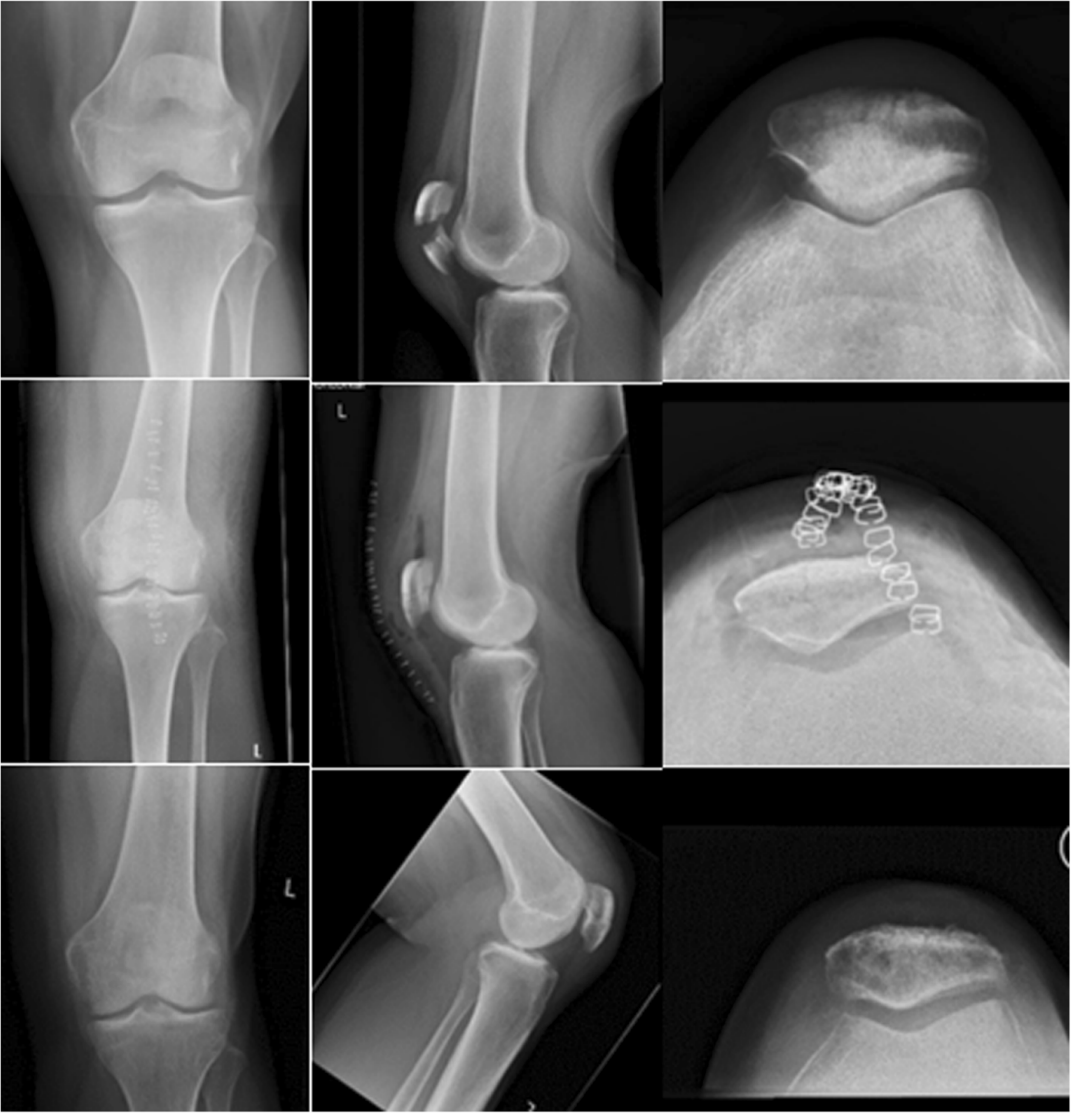
Pre-, post-, and 6-month postoperative X-rays (above, middle and below, respectively) of patient 6, a 58-year-old woman with a simple transverse patella fracture

All six patients had fractured their patella following a simple fall during normal daily activities (Figs. 6 and 7). Two and four fractures were simple and comminuted transverse, respectively, and none were classified as open fractures.

At the 6-month follow-up, all six cases were radiologically and clinically assessed. One patient (patient 3) had a minor loss of reduction (<2 mm) but did not require re-operation and had no functional deficit at the final follow-up. The remaining five patients were united with the intra-operative anatomic reduction. No patients required re-operation related to their fracture or sutures. No patients had wound complications or infections. Whilst no patients could feel the sutures or knots at the final follow-up, four patients had altered sensation around the surgical scar.

Clinical assessment performed at the 6-month follow-up showed normal extension and maximal flexion of 100 degrees in all six patients. The Lysholm knee score was used to assess the knee function. The score has eight items related to activities of daily living and ranges from zero to 100 points, with a higher score indicating fewer symptoms and higher level of functioning [15]*.* The Lysholm score was 90 points or more in five of the six cases. The last patient (patient 4) had a score of 78 points. No patients experienced giving way or locking of the knee and all were fully ambulatory without the need for crutches at the final follow-up. None of the six patients had pain at rest or during activity (VAS 0). Five of the six patients (except patient 1) reported problems climbing stairs, especially descending, at the final follow-up, probably due to a lack of quadriceps strength. Three of the six patients (patients 1, 2, and 3) had no problems squatting. Two of the remaining three had slight problems, whilst the last (patient 4) was not able to squat.

## Discussion

Surgical treatment of patella fractures is challenging, with reoperation rates following internal fixation as high as 52%, mostly due to irritation from prominent metallic implants [12, 14]. To overcome implant irritation, we present a standardised technique for the internal fixation of both simple and comminuted patella fractures using braided sutures. Patella fixation with sutures is not a novel technique as several have described small case series of various techniques. With our technique, we describe the technique detailed and the technique adapts to different fracture configurations. Furthermore, this technique bypasses the bone which is the weakest point. None of the six patients experienced prominent sutures or knots leading to irritation. We believe this technique is a safe and promising alternative mean of fixation.

The strength of the braided sutures used for this technique has been tested biomechanically and was a strong choice for implant when pulled in a straight line, maintaining initial stiffness until failure and may be superior to stainless steel cerclage in transverse patella fractures [16–18]. We did not experience any fracture displacement due to suture tear.

We highlight the following six technical tips to achieve successful results with this technique (Table 1).

**Table 1:** Six technical tips for the suture tension band fixation technique

1. Meticulously clear all debris and contused tissue to avoid secondary infection. 2. Reconstruct the patella fragment by fragment and use k-wires to hold the reduction. Use only sutures to hold the achieved reduction. 3. The sutures passed through the quadriceps and patella tendon should be as close to bone as possible to avoid that the sutures secondarily cut through the tendon resulting in slack and potentially displacement. 4. The sutures passed through the quadriceps and patella tendon should grasp the tendons as deep as possible to hold the reduction at the articular surface. 5. Using the “modified circular and figure-of-eight” sutures, a minimum of four strings will cross the anterior surface of the patella, providing a “basket” to contain the reduction of the fragments. 6. The knots should be tied on the lateral aspect of the patella and never on the anterior aspect of the patella to avoid irritation from prominent knots.	

The limitations of this study include the lack of comparison to previous series as this study is presented as a technique. Furthermore, the small case series and the fact that all patients were treated by a single surgeon at a single centre are also potential limitations.

A recent review on non-metallic fixation of patella fractures reported a success rate of 90% in 123 patella fractures, with additional surgery for implant removal required for only four patients (3.2%). In this review, the nine included studies described nine different fixation methods using sutures, bioabsorbable screws/plugs, or a combination of the two. Most of the studies had a selection bias regarding the fracture type [19]. Indeed, non-metallic fixation techniques for patella fractures are not novel and have been used in various configurations and have likewise been biomechanically tested and seem clinically promising [20, 21].

A concern we have with the described technique is the amount of foreign materiel used to treat comminuted fractures. With the most comminuted fractures, we advise to use four sutures all knotted on the lateral border of the patella. If this number of sutures increases the risk of infection is unknown and must be investigated. But compared to the amount of metallic implants (wires, k-wires, and screws) used for comminuted fractures, we believe the load of foreign implants is acceptable. We believe that the suture tension band fixation technique is suitable for all types of patella fractures due to the ‘basket’ configuration achieved by the many crossing suture strings in addition to the step-wise standardised technique that can be adapted to the fracture configuration.

## Conclusions

The suture tension band fixation technique described in details in the present report is an alternative treatment for patella fractures that may reduce the rate of prominent implants. The technique was described in a step-wise, standardised way and can be adapted to all types of patella fractures.

### Limitations

A small number of cases, however, in this study, are a descriptive technical note.

This technique has only been used by a limited number of surgeons.

## Data Availability

Data sharing is not applicable to this article as no datasets were generated or analysed during the current study.

## Abbreviations

STB: Suture tension band
ROM: Range of motion
VAS: Visual analogue score
